# Aging-associated increase in indoleamine 2,3-dioxygenase (IDO) activity appears to be unrelated to the transcription of the IDO1 or IDO2 genes in peripheral blood mononuclear cells

**DOI:** 10.1186/1742-4933-8-9

**Published:** 2011-10-11

**Authors:** Saara Marttila, Juulia Jylhävä, Carita Eklund, Antti Hervonen, Marja Jylhä, Mikko Hurme

**Affiliations:** 1 Department of Microbiology and Immunology, The School of Medicine, University of Tampere, Finland; 2 The School of Health Sciences, University of Tampere, Finland; 3 Centre for Laboratory Medicine, Tampere University Hospital, Finland

## Abstract

**Background:**

Old age is associated with increased levels of circulating pro-inflammatory cytokines, a phenomenon termed inflamm-aging. Elevated levels of pro-inflammatory cytokines have been associated with several age-associated diseases and with a shortened lifespan. Indoleamine 2,3-dioxygenase (IDO) has immunomodulatory properties and its activity is elevated in inflammation, autoimmune disorders and malignancies. We have previously shown that IDO activity is increased in nonagenarians compared to young individuals and that high IDO activity is associated with mortality at old age.

**Findings:**

In this study our aim was to assess whether this difference in IDO activity in the plasma was due to the differential expression of either the IDO1 or IDO2 gene in peripheral blood mononuclear cells. Our results show that IDO1 and IDO2 are not differently expressed in nonagenarians compared to controls and that the expression of IDO genes is not associated with the level of IDO activity in the plasma.

**Conclusion:**

The level of IDO activity in the plasma is not regulated through the expression of IDO1 or IDO2 in the peripheral blood mononuclear cells.

## Findings

The aging-associated decline of the immune system, termed immunosenescence, is characterized by aberrantly functioning T cell populations and an increased level of circulating pro-inflammatory cytokines (inflamm-aging). The levels of CRP, IL-6, TNF-α, among others, are increased in the blood of aged individuals and this increase is associated with a shortened lifespan [1,2]. The high levels of pro-inflammatory cytokines are also associated with several age-related conditions such as dementia, Parkinson's disease, atherosclerosis, type 2 diabetes, sarcopenia and functional disability. An age-associated increase in the production of TNF-α, IL-6 and IL-1Ra has been reported in unstimulated peripheral blood mononuclear cells (PBMCs). However, other cell types, such as endothelial, adipose and macrophage-derived cells, probably also contribute to the plasma levels of these and other pro-inflammatory cytokines. The inducers of these molecules and the mechanisms of activation of the genes associated with them remain poorly characterized [1,2].

Indoleamine 2,3-dioxygenase is an immunomodulatory enzyme, the activity of which is elevated in several inflammatory conditions, such as infection, autoimmune disorders and malignancies [3]. The IDO enzyme is the first and also the rate-limiting enzyme in the pathway that converts tryptophan (trp) to kynurenine (kyn). IDO can suppress effector T cells and stimulate the differentiation of naïve T cells to regulatory T cells (Tregs). The levels of IDO are elevated when the immune reaction is polarized towards the Th1 response, but there is also evidence of a negative feed-back loop, where high IDO levels down regulate the Th1 response and stimulate a Th2 response [4,5].

There are two genes encoding IDO enzyme, IDO1 and IDO2. IDO1 is expressed intracellularly in the placenta, lung, small intestine, colon, spleen, liver, kidney, stomach and brain. Cell types that express IDO1 include myeloid-lineage cells (dendritic cells, monocytes, macrophages and eosinophils), epithelial cells, fibroblasts, vascular smooth muscle cells, endothelial cells and certain tumor cell lines. The main inducer of IDO1 both *in vitro *and *in vivo *is type II interferon (IFN-γ), but it can also be induced by IFN-α, IFN-β and lipopolysaccharide (LPS). Other factors can also modulate IDO1 expression depending on the cell type [6]. IDO2 was only recently found, and its biological role is still unclear. In humans IDO2 mRNA is expressed in the liver, small intestine, spleen, placenta, thymus, lung, brain, kidney and colon [7]. It appears that the enzyme produced by IDO2 can catabolize the same substrates as IDO1 but with lower efficiency [8]. Mouse studies show that when IDO1 and IDO2 are expressed in the same tissue, they are expressed in different cell types, suggesting that there is no functional redundancy between the two enzymes [9]. The hepatic enzyme tryptophan 2,3-dioxygenase (TDO) also catalyzes the same reaction as IDO, but there are no indications that it is regulated by inflammatory cytokines. However, as aging affects the majority of organ systems, the contribution of TDO to the elevated degradation of trp cannot be completely excluded.

The ratio of first metabolite, kyn, to the substrate, trp, can be used as an indicator of IDO activity in circumstances related with concomitant immune activation. Increased degradation of trp has been associated with elevated levels of inflammation markers, such as neopterin and IFN-ɣ, in HIV infection, sepsis, autoimmune disorders and malignancies [3]. The same phenomenon is seen also in the elderly population, in which increased degradation of trp is associated with increased production of homocysteine and neopterin [10].

The association of inflammation and IDO activity in the plasma has been shown in various studies, but the majority of these studies did not identify the cell type producing the IDO enzyme. However, in untreated HIV infection the level of trp degradation i.e. IDO activity has been shown to be elevated [11,12] and Boasso et al. have shown that the level of IDO mRNA expression in the PBMCs has also increased [13].

We have shown that the activity of IDO in the plasma, as determined by the ratio of the main metabolite to substrate, kyn to trp, is increased in nonagenarians compared to young individuals, and that the high IDO activity is associated with a shortened lifespan in nonagenarians during a four-year follow-up [14]. In this study our aim was to assess whether the difference of IDO activity in the plasma was due to the differential expression of either IDO1 or IDO2 in peripheral blood mononuclear cells. This is the first study assessing the relationship of IDO expression and IDO activity in a non-stimulated and non-acute situation.

The study population consisted of 12 healthy women (nonagenarians, n = 8 and controls, aged 25-37, n = 4). The nonagenarian women represented the best-functioning respondents to the mailed Vitality 90+ survey. For original cohort description, see [15]. The blood samples were drawn by a home-visiting nurse into EDTA collection tubes and were directly subjected to leukocyte separation with Histopaque 1077 density gradient (Histopaque^®^-1077, cat. no. 10771, Sigma-Aldrich, MO, USA). The plasma layer was collected and stored at -20°C. The PBMC layer was collected, and the cells were suspended into 1 ml of RPMI-160 medium (cat. no. R0883, Sigma-Aldrich, MO, USA). Immediately after the PBMC separation, traces of erythrocytes were lysed with a 10-second H_2_O treatment and immediately recovered with 0.9% NaCl. Plasma concentrations of trp (mmol/L) and kyn (μmol/L) were measured with high-performance liquid chromatography as described previously [16]. Briefly, samples were deproteinized with nitrotyrosine containing TCA-buffer. Samples were mixed troughoutly and incubated at +4°C for 15 minutes, after which they were centrifuged twice with 10000 × g for 15 and 6 minutes. The clear supernatant was used for the analysis. Trp was separated with a Shimadzu liquid chromatograph LC-10AD VP (Shimadzu Co, Kyoto, Japan) using a 50-mm DS HypersilC18 5 μm column (Thermo Electron Co, Bellefonte, PA, USA) and was monitored with a Shimadzu RF-10A XL detector at 266 nm excitation and 366 nm emission wavelengths. Kyn was separated with a Hewlett-Packard 1100 liquid chromatograph (Palo Alto, CA, USA) using LiChroCart 55-4150 mm cartridge containing a Purospher STAR RP-18 3 μm column (Merck Co, Darmstadt, Germany) and was analyzed at 360 nm wavelengths with a Hewlett-Packard G13144 detector. Total RNA extraction from PBMCs was performed with the Qiagen RNeasy^® ^Midi kit (cat. no. 75144, Qiagen, CA, USA) according to the manufacturer's instructions. Levels of IDO1 and IDO2 transcripts in the PBMCs were determined with TaqMan real-time PCR using separate gene expression assays (Hs00158027_m1 for IDO1 and Hs00401201_m1 for IDO2, Applied Biosystems, CA, USA). To determine whether IDO1 or IDO2 are expressed differently between the nonagenarians and the control population, we calculated the RQ value for those genes. This was done with Relative Quantification (RQ) documents and the RQ Manager Software (Applied Biosystems, CA, USA).

**Figure 1:**
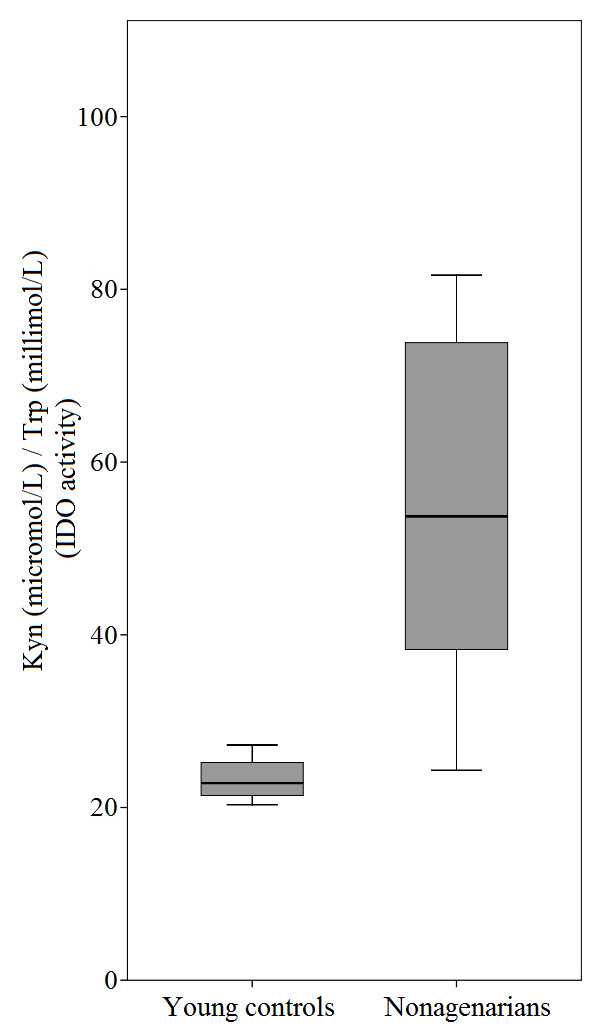
**IDO enzyme activity in nonagenarians and young controls**. The ratio of kyn to trp, indicating IDO enzyme activity, is elevated in the plasma of nonagenarians compared to young controls (p = 0.011, Mann-Whitney U-test).

**Figure 2:**
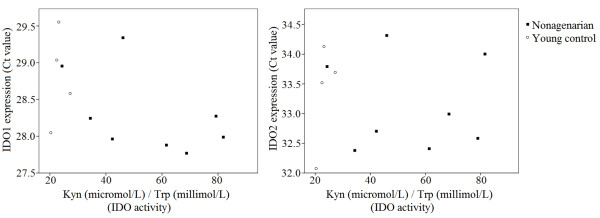
**IDO activity and expression**. The level of IDO enzyme activity in the plasma does not correlate with the level of IDO1 or IDO2 expression in PBMCs in the whole population (Spearman's rho -0.503, p = 0.095; rho = 0.077 p = 0.812, respectively), nor in the nonagenarians or young controls separately.

As in our previous work [14], there was a significant difference in the IDO activity in the plasma between the nonagenarians and the young controls; the level of IDO activity is higher in the plasma of the nonagenarians compared to young controls (Figure 1). However, the data obtained showed that there was no difference in the expression levels of IDO1 and IDO2 in nonagenarians compared to young controls (Table 1). Neither did the level of IDO1 or IDO2 expression in PBMCs correlate with the IDO activity in the plasma in nonagenarians or in young controls (Figure 2).

**Table 1:** The expression levels of IDO1 and IDO2 in PBMCs.

	ΔCt Controls	ΔCt Nonagenarians	RQ	p
IDO1	4.82	5.05	0.85	n.s.
IDO2	9.17	9.75	0.67	n.s.

No statistically significant differences were found in the expression levels of IDO1 or IDO2 in PBMCs between nonagenarians and young controls, despite the difference in the level of IDO enzyme activity in the plasma.

Our data indicates that the level of IDO enzyme activity in the plasma is not produced by the expression of IDO1 or IDO2 in PBMCs. PBMCs isolated by density centrifugation consist mainly of T cells, the proportion of monocytes, macrophages and dendritic cells is smaller. However, the latter cell types might be more important for IDO activity. Boasso et al. [13] showed that only 3% of PBMCs express IDO, majority of these were plasmacytoid dendritic cells. However, the difference in IDO mRNA expression observed in their study between patients and controls was seen in uncharacterized PBMCs. Thus if the difference in IDO activity in the plasma was produced even by a small subpopulation of PBMCs, the difference in IDO1 or IDO2 gene expression should have been detected also in our sample of uncharacterized PBMCs.

The high concentrations of kyn compared to trp in the plasma of nonagenarians probably is a result of IDO expression in other cells and tissues aside from PBMCs. Local IDO activity has been shown to affect systemic trp concentrations, at least in malignancies [17]. One strong candidate for the source of IDO activity in the plasma is the IDO expression in the endothelial cells of blood vessels [18,19]. It is also possible that the level of IDO activity is regulated through the posttranslational modifications that are needed for an active IDO enzyme, for example, the binding of heme [20]. It is of interest whether the elevation of IDO activity observed in different inflammatory states is brought about by different cell types depending on the case, especially if there is a difference between inflamm-aging and other inflammatory states.

## Competing interests

The authors declare that they have no competing interests.

## Authors' contributions

SM performed the experiments and the statistical calculations as well as drafted the manuscript. JJ performed the experiments and helped to draft the manuscript. CE performed the experiments. AH and MJ recruited the study participants. MH provided the reagents and materials for the study, designed the study and helped to draft the manuscript. All authors read and approved the final manuscript.
